# A Modified Left Ileal Conduit in the Presence of a Left Short Ureter Following an Urgent Radical Cystectomy: A Case Report

**DOI:** 10.7759/cureus.42033

**Published:** 2023-07-17

**Authors:** Hakan Çakıcı, Atınç Tozsin, Gökhan Çevik, Hakan Akdere

**Affiliations:** 1 Urology, Optimed Hospital, Tekirdağ, TUR; 2 Urology, Trakya University Hospital School of Medicine, Edirne, TUR; 3 Urology, Sultan 1. Murat State Hospital, Edirne, TUR

**Keywords:** uretero-ileal anastomosis, bladder cancer, urinary diversion, radical cystectomy, ileal conduit

## Abstract

The ileal conduit is the most common method performed for urinary diversion following radical cystectomy. The prepared conduit is usually placed on the right abdominal wall. There is not enough experience and literature on left-sided ileal conduits. Here, we report a case of a left-sided ileal conduit with a modified method and describe the surgical technique. A 68-year-old male patient had undergone an urgent radical cystectomy operation one year ago due to bladder cancer and gross hematuria. However, urinary diversion was not performed, and a bilateral nephrostomy was inserted. An ileal conduit was planned for the patient after oncological stabilization. On preoperative evaluations, bilateral ureters were observed to be short, with the left being prominent. The prepared ileal conduit was passed under the sigmoid mesentery due to the short ureters and placed on the left abdominal wall. There were no major complications during follow-ups. We emphasize that the method we performed is a safe option in mandatory situations.

## Introduction

Radical cystectomy is recommended for muscle-invasive bladder cancer. The ileal conduit is the most common method for urinary diversion with well-known or predictable outcomes following radical cystectomy [1]. The prepared conduit is usually placed on the right abdominal wall. There is not enough experience and literature on the left-sided ileal conduit [2,3]. Here, we report a case of left-sided ileal conduit with a modified method and describe the surgical technique.

## Case presentation

**Figure 1:**
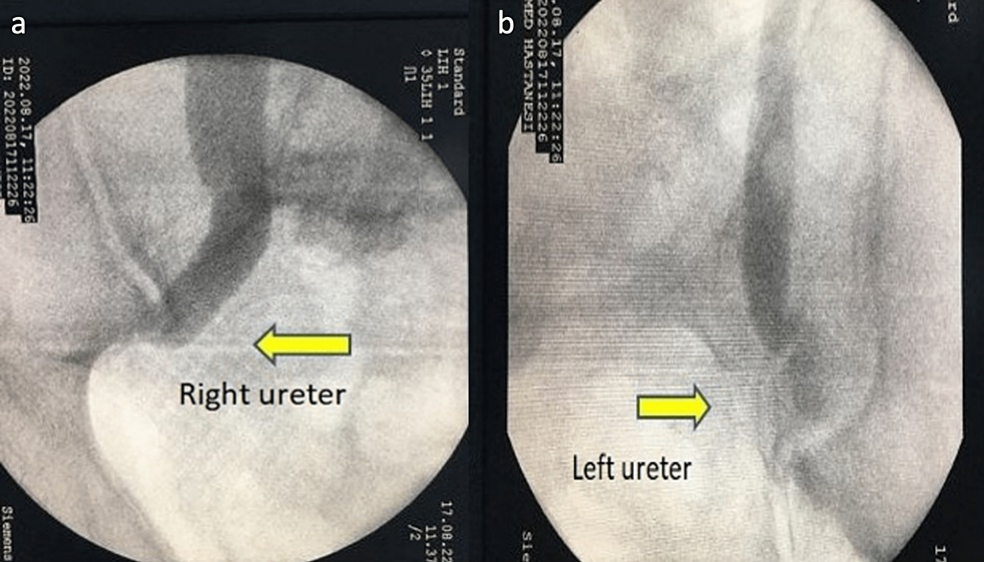
Antegrade pyelography. a. Right ureter. b. Left ureter.

A 68-year-old male patient had undergone an urgent radical cystectomy operation one year ago. Due to the patient being in a foreign country and the lack of an available medical summary, a comprehensive medical history could not be obtained. An emergency radical cystectomy was performed due to uncontrolled gross hematuria that was unresponsive to conservative treatment. However, due to limited access to the patient’s medical history and lack of information, we were unaware of the clinical condition and specific laboratory values at the time of the surgery. Given the absence of a comprehensive medical record, it is uncertain why a urinary diversion was not performed and a nephrostomy was placed instead. We suspect that this decision was made due to perceived peroperative instability. The patient was admitted to our clinic due to recurrent infections caused by nephrostomy and deteriorated quality of life. There were no available postoperative pathology reports or any records regarding the oncological treatment received by the patient. Upon reviewing the contrast-enhanced CT, it was determined that the patient was metastasis-free, leading to the decision to proceed with an ileal conduit. Preoperative hemoglobin was 10.4 g/dL (normal = 12.3-17.5 g/dL), urea was 134 mg/dL (normal = 10-50 mg/dL), and creatinine was 5.1 mg/dL (normal = 0.5-1.1 mg/dL). The patient consulted with the nephrology department. However, renal replacement therapy was not considered as there was no evidence of electrolyte imbalance. Bilateral ureters (more prominent on the left) were observed to be short on preoperatively performed antegrade pyelography (Figure 1), and we planned to perform a left ileal conduit.

**Figure 2:**
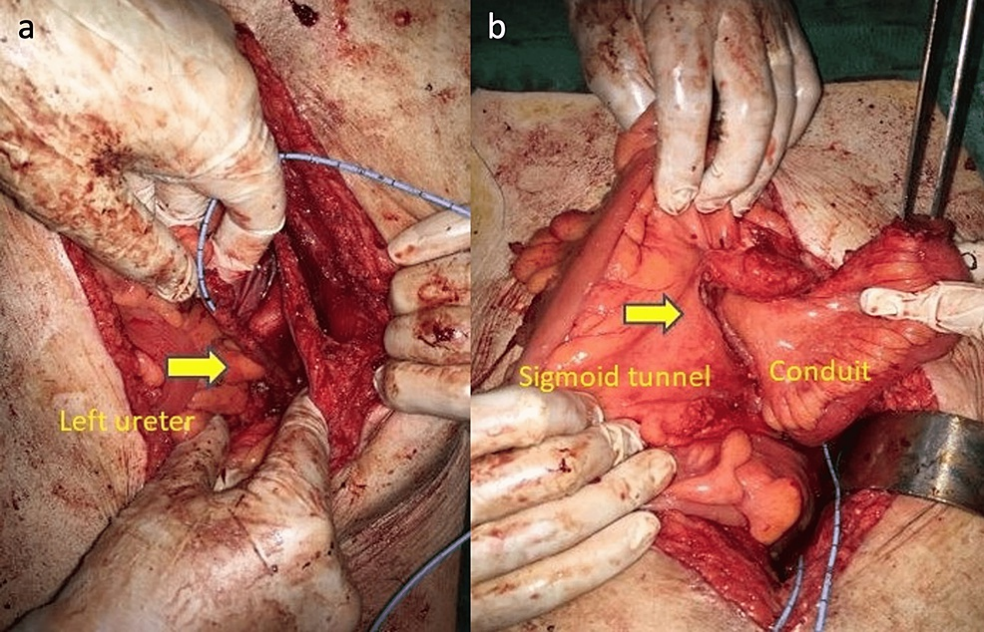
The tunnel formed under the sigmoid mesentery. a. Left ureter. b. Mesenteric tunnel and ileal conduit.

After the right and left ureters were dissected, the left ureter was observed to be significantly short. Additionally, the right ureter was shorter than normal. Distal fibrotic ureteral tissues were excised, and a frozen section was performed. No malignancy was detected. A 20 cm long ileum segment was prepared for the ileal conduit. The proximal part of the ileum was prepared for ureteral anastomoses and the distal part for the stoma. Due to the possibility of developing intestinal adhesions as a result of tertiary open surgery and the likelihood of encountering atypical anatomy as a result of the use of a modified approach, it was decided to consult with the general surgery team for intraoperative appendectomy if a potential appendicitis were to occur in the patient’s later life. During the intraoperative evaluation, it was thought that passing the right ureter and ileal conduit to the left side under the sigmoid colon would be more suitable for tension-free and robust anastomosis. The right ureter and the prepared ileal conduit were passed to the left side through the tunnel formed under the sigmoid mesentery (Figure 2).

**Figure 3:**
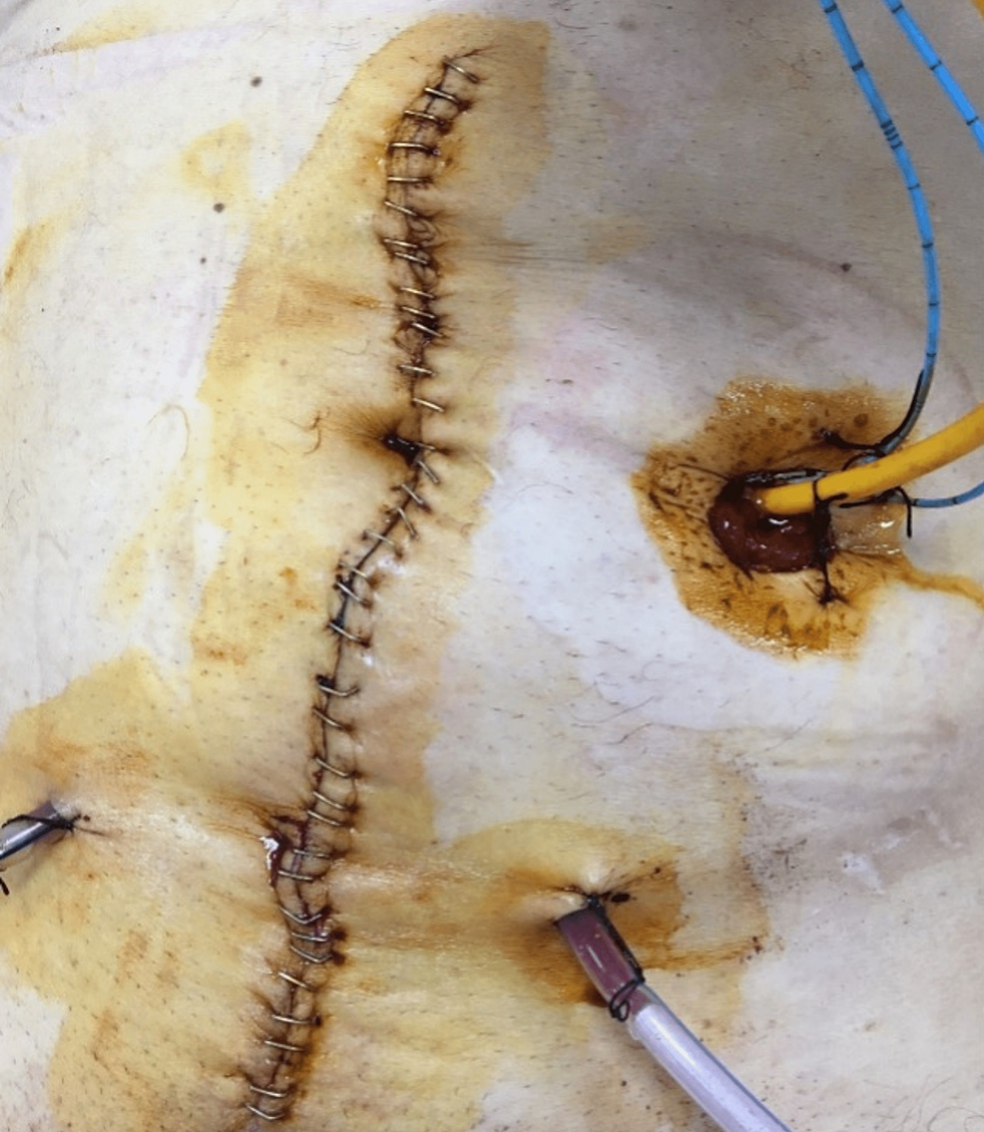
Ileal conduit placed on the left abdominal wall.

Mono-J catheters were inserted into the ureters and ureteral anastomoses were performed using the Bricker method. The ureteral anastomoses were placed in the retroperitoneum by making an incision in the posterior peritoneum and the peritoneum was closed over them. The distal part of the conduit was taken out from the rectus abdominis muscle and placed on the left abdominal wall (Figure 3).

A Foley catheter was inserted in the conduit. There were no major complications in the postoperative follow-up. The Foley catheter was removed on the second and Mono-J catheters were removed on the 10th postoperative day. The patient was discharged on the 14th day. In the last laboratory tests, hemoglobin was 10.8 g/dL, urea was 72 mg/dL, and creatinine was 3.6 mg/dL.

## Discussion

The ileal conduit after radical cystectomy is usually placed on the right abdominal wall. Because of the anatomical convenience and peristalsis, the right side is preferred. However, it may not be possible to place an ileal conduit on the right abdominal wall in mandatory situations. There is not enough experience and literature on the left-sided ileal conduit. Nakamura et al. placed a stoma on the left side in a patient with a hernia in the right abdominal wall [2]. In our case, due to the short ureters, the prepared ileal conduit was passed through the tunnel prepared by us under the sigmoid mesentery and placed on the left abdominal wall. As Kotb et al. reported the modified technique, the ileal conduit was passed under the sigmoid mesentery with no reported complications [3]. Li et al. reported successful results by passing the ileal conduit under the sigmoid mesentery using their modified technique [4]. In our opinion, taking care not to bend the conduit’s mesentery and the sufficient width of the prepared tunnel are the main goals to be considered. Although we preferred the Bricker method for uretero-ileal anastomosis, we think that the Wallace method could also be performed if the ureters were of sufficient length [5,6].

## Conclusions

We report a case where a patient with a left ileal conduit passed under the sigmoid mesentery. We emphasize that our method is a safe option in mandatory situations.
